# Intestinal microbiota, probiotics and mental health: from Metchnikoff to modern advances: part III – convergence toward clinical trials

**DOI:** 10.1186/1757-4749-5-4

**Published:** 2013-03-16

**Authors:** Alison C Bested, Alan C Logan, Eva M Selhub

**Affiliations:** 1 Complex Chronic Diseases Program, BC Women’s Hospital and Health Centre, B223A-4500 Oak Street, Vancouver, BC, V6H 3N1, Canada; 2 CAMNR, 775 Blithedale Avenue Suite 364, Mill Valley, CA 94941, USA; 3 Harvard Medical School and Massachusetts General Hospital, 40 Crescent St., Suite 201, Waltham, MA, 02453, USA

**Keywords:** Intestinal microbiota, Autointoxication, Depression, Anxiety, Probiotics, Microbial ecology, Lipopolysaccharide endotoxin, Diet, Intestinal permeability, MMP-9, Microbial ecosystems

## Abstract

Rapid scientific and technological advances have allowed for a more detailed understanding of the relevance of intestinal microbiota, and the entire body-wide microbiome, to human health and well-being. Rodent studies have provided suggestive evidence that probiotics (e.g. lactobacillus and bifidobacteria) can influence behavior. More importantly, emerging clinical studies indicate that the administration of beneficial microbes, via supplementation and/or fecal microbial transplant (FMT), can influence end-points related to mood state (glycemic control, oxidative status, uremic toxins), brain function (functional magnetic resonance imaging fMRI), and mental outlook (depression, anxiety). However, despite the advances in the area of gastro-biological psychiatry, it becomes clear that there remains an urgent need to explore the value of beneficial microbes in controlled clinical investigations. With the history explored in this series, it is fair to ask if we are now on the cusp of major clinical breakthroughs, or are we merely in the quicksand of Autointoxication II?

## Introduction

Though the first two parts of this series, we have attempted to provide a historical and contextual approach to the more direct lines of contemporary evidence as it relates to gut microbiota, its intentional manipulation, and mental health. Here in part III we will discuss more specific research related to the direct experimental and clinical effects of probiotics in the context of stress, neurophysiology, behavior and mental outlook. The emerging studies clearly show that we are in the midst of a very exciting time within gastro-biological psychiatry. Still, as we conclude our series, it becomes evident that there many unanswered questions and an urgent need for expanded clinical investigations.

### Experimental dysbiosis, stress and probiotics

More clinically relevant information related to behavior is provided by animal studies involving interactions between stress, dietary habits and alterations to the normal homeostasis of gut microbiota (i.e. dysbiosis rather than germ-free). The term dysbiosis is often erroneously attributed to Metchnikoff, however, both eubiosis (healthy life in steady state) and dysbiosis (shift from healthy state of being) were already in popular use decades before Metchnikoff gained fame. Physician Elliot E. Furney used the terms together in his 1890 book related to plant, animal and human resiliency [1]. In the context of intestinal microbiology, dysbiosis is a term that gained medical acceptance in the 1960s [2]. In animal models, researchers can induce dysbiosis through experimental infection, psychological stress, Western dietary habits, and/or the administration of antimicrobial agents [3]. To date, the emerging research shows that all of these factors may interact together in a vicious cycle – i.e. in experimental studies, induced states of dysbiosis can promote behavior indicative of anxiety and cognitive dysfunction in animals [3]. On the other hand, psychological stress itself leads to dysbiosis [4,5], and it also encourages the consumption of a fast-food-style diet [6], which only serves to further promote dysbiosis [7].

More directly, however, the anxious-like behavior of dysbiosis invoked by small amounts of gut pathogens is associated with increased markers of activation within limbic structures of the brain (including the amygdala) [8]. Intestinal vagal activity is also increased with pathogenic dysbiosis, adding yet more evidence that the vagus nerve is a primary portal of communication from intestinal microbiota upward toward the brain [9]. Induction of low-grade gastrointestinal inflammation causes anxiety-like behavior in animals; however in animals with a prior vagotomy, the behavior disturbances associated with gut inflammation were not evident. Moreover, the oral administration of the probiotic *Bifidobacterium longum* NCC3001 (Morinaga, Japan) prevented the anxiety-like behavior associated with gut inflammation in animals with an intact vagus nerve, but it did not have this anxiolytic effect if the vagotomy was performed after chronic low-grade inflammation. Remarkably, the reduction in anxiety-like behavior after probiotic administration was not associated with any detectable reduction in gut inflammation *per se*[10]. In other words, the vagus nerve appears to be a conduit for behavioral change - anxiogenesis (via gut inflammation) and anxiolysis (via the presence of a probiotic). Separate research involving a strain of *Lactobacillus rhamnosus* also showed that administration to healthy animals in stressful situations reduced anxiety and depression-like behaviors in the elevated plus maze (EPM) and forced swim tests. These behavioral changes were linked to alterations in the gamma-aminobutyric acid (GABA) system of the brain in the probiotic group, yet the changes in behavior and brain chemistry were largely extinguished with vagotomy [11].

As we argued almost a decade ago regarding depression and the gut-brain axis, some species of intestinal microbes can make significant contributions to the prodcution of GABA and other neurotransmitters in vivo [12]. The extent to which this local GABA intestinal production can influence systemic physiology, depression and anxiety remains a mystery [13]. For now, we do know through preliminary research that the oral administration of GABA derived from *Lactobacillus hilgardii* fermentation has been shown to have clinical value in anxiety reduction [14]. As for vagus communication, the vagal innervations of the intestinal tract are rich in the small intestine, with relatively fewer in fibers extending into the large intestine. Therefore it is also noteworthy that other studies have shown that prior vagotomy does not always prevent anxiety-like behavior in the presence of gut pathogens [15]. This suggests that there are multiple lines of communication between the intestinal tract and brain. Indeed, the administration of *Bifidobacterium longum* normalized anxiety-like behavior and hippocampal BDNF levels among animals when the parasite *Trichuris muris* was introduced orally – the behavioral benefits were once again independent of inflammatory cytokines [15].

As described in Part I, Lloyd Arnold reported in the 1920-30s that dietary context can influence intestinal-pathogen-induced symptoms and mortality. Remarkably, he found that the standard stock rodent chow, one that they had been using for years in his lab to raise mice, was associated with the highest mortality in mice upon *Salmonella enteritides* exposure vs. other natural foods. In particular, a switch from the standard chow to banana powder decreased mortality from 96% to 6% over one month post-infection [16]. For Arnold, the food vehicle was an important factor in intestinal microbiology outcomes. Although he did not determine it at the time, modern research shows the banana to promote the growth of bifidobacterium [17]. When contemporary researchers added 97% lean meat to the standard diet of rodents for 3 months they found an increase in the diversity of the intestinal microbiota, as well as decreased behavioral signs of anxiety and improved aspects of cognition [18]. From the experience of Arnold, we cannot assume that the “standard” lab chow is the default healthy fare. Although this type of research on meat added to standard fare establishes correlation and not causation, it should provide a stimulus to more closely examine the potential links between Paleolithic nutrition, of which lean meat was a significant part, its reported health benefits [19], and the diversity of the intestinal microbiota.

Of course, this research also exposes the short-comings of the term dysbiosis, in that a shift from ‘normal’ homeostasis of intestinal microbiota (via a better quality diet) might actually confer *benefit* to the host. The same is true of antimicrobial administration – clearly, antimicrobial agents can cause massive shifts in gut microbiota, yet here again the oral administration of these agents to mice (anxious BALB/c strain) caused a decrease in anxiety-like behavior. Such was not the case when the antimicrobial agents were delivered by injection, nor were there any behavioral changes when the agents were delivered orally to germ-free mice [20]. As mentioned, the regular consumption of a high-fat diet causes alterations in the characteristics of the intestinal microbiota, yet when antimicrobials are administered along with the Western-style diet, the detrimental diet-induced effects on systemic inflammation, systemic LPS burden and glucose-insulin dysregulation are minimized [21].

It is unlikely that antibiotics are a long-term solution to depression; however, the recently reported success of minocycline in depression [22] can no longer be theorized to simply be a matter of the direct neuroprotective properties of the agent. Together, the combination of minocycline and probiotic (*E. coli Nissle* 1917) is more effective at reducing experimental intestinal inflammation and markers of intestinal permeability vs. either one alone. In particular, this combination of probiotic and antibiotic significantly reduced the expression of matrix metalloproteinase 9 (MMP-9) [23], a salient finding given the emerging research suggesting that increased MMP-9 activity is associated with loss of intestinal microbiota diversity. In the period following a challenge with pathogenic intestinal microbes, MMP-9 knockout mice show an increase in lactobacilli [24], meanwhile increased MMP-9 activity is associated with intestinal permeability [25,26], and the severity of human depression [27-29]. Since consumption of the Mediterranean diet is associated with decreased MMP-9 activity [30], researchers must once again attempt to decipher the extent to which dietary benefits might be intertwined with intestinal microbiota. When a multi-strain probiotic is consumed with a high fat (predominantly corn oil) diet, the elevations of MMP-9 and systemic oxidative stress is minimized [31].

### Alvarez revisited, again

Some of the more intriguing animal studies in recent years have involved the efficacy of probiotics in the reduction of visceral pain, including that related to colorectal distention. One study involved 9 days of probiotic supplementation (*Lactobacillus reuteri* vs. control) prior to experimental colorectal distension in rats. Remarkably, live probiotic prevented the physiological signs of visceral pain (marked bradycardia) that was otherwise evident in the control group. The researchers found the same effect whether or not the *L. reuteri* was alive, heat or radiation-killed, indicating that even structural components of probiotics can play a role in visceral sensitivity. This reduction in cardio-autonomic response in the animals was noted even at the highest level of distention pressure (80mm Hg), and the resiliency was accompanied by an inhibitory effect on discharge of colonic afferent nerve fibers in the probiotic groups [32]. Separate research has found that the oral administration of *Bifidobacterium infantis* 35624 can reduce visceral pain behaviors in both normosensitive and hypersensitive-anxious rat strains during colorectal distention [33]. The same strain of *Bifidobacterium infantis* can normalize sensitivity to colorectal distention in a rat model of post-inflammatory colonic hypersensitivity [34]. Moreover, *Bifidobacterium lactis* CNCM I-2494 in a fermented dairy matrix has been shown to reduce visceral hypersensitivity when colorectal distention occurred in the context of psychological stress. This study also showed that the probiotic reduced intestinal permeability and blood endotoxin levels. It is noteworthy that the antinociceptive effect of the fermented dairy product (inclusive of the aforementioned bifidobacterium strain plus *Lactococcus lactis* and two yogurt starters *L. bulgaricus and S. thermophilus*) was more effective than an equivalent dose of *Bifidobacterium lactis* CNCM I-2494 alone [35]. Here we see the relevance of the often overlooked interplay of beneficial microbial synergy and the context of the food matrix.

*Lactobacillus farciminis* has also been shown to abolish colorectal distension hyeralgesia in an animal model of acute stress. Indeed, the expression of Fos, a marker of general neuronal activation, was activated in limbic pathways (paraventricular nucleus of the hypothalamus, medial nucleus of the amygdala) by colorectal distention – however, *L. farciminis* administration significantly reduced Fos expression in these areas during colorectal distention [36]. Finally, researchers have also shown that antibiotic-induced shifts in intestinal microbiota increases visceral hyperalgesia in healthy mice, and that the administration of *Lactobacillus paracasei* NCC2461 prevented this antibiotic-induced visceral sensitivity [37]. Importantly, the administration of *L. paracasei* did not restore normal lactobacilli levels overall, indicating that a major influence on gut microbial genera is not required for therapeutic efficacy via probiotics.

Although these are preliminary animal studies, one cannot help but to reconsider the influential voice of intestinal toxemia’s most vehement critic. Recall from Part I that Alvarez, who focused his attention on Freudian-inspired conditions - ‘hysterical’ bloating and ‘neurosis of the abdominal wall’ as he called them - claimed that personal (non-immune, non-microbial) sensitivity to distention was at the heart of the gastrointestinal complaints in many of those with neurasthenia. Alvarez turned out to be correct in proposing a psycho-mechanical theory, his 1919 assertion [38] that ‘*in sensitive people the brain is profoundly influenced by afferent impulses coming from a distended, underactive or unduly active bowel*’ – certainly modern day interoseptive sensitivity in anxiety and pain-related conditions would support this notion [39,40]. However, microbes and their breakdown products, their local and systemic effects in mental outlook and pain, are clearly emerging as part of the equation. Could microbes be a bottom-up player in that personal sensitivity? Might microbes be responsible for neurosis of gut linings and hysterical bloating? The above research on probiotics and colorectal distention will force scientists to explore alternative research pathways.

Consider the separate research on methane producing microbes within the intestines; these microbes have been consistently linked to constipation [41-43], and are now thought to directly mediate motility via methane production [44]. Recent investigations demonstrating small intestinal bacterial overgrowth (SIBO) and excess methane production in children with encopresis might suggest that the condition may not be exclusively psychogenic [45]. Constipation is 5 times more likely in children with the related condition of nocturnal enuresis [46], and intestinal pathogens have been associated with increased risk of enuresis in children [47]. As lines of investigation become integrated, it may be worth examining how might probiotics mediate pain and childhood behavioral disorders via their interaction with methane-producing and other living intestinal residents.

### Putrefactive chemicals – still an open question

A primary focus among the scientists and clinicians of the autointoxication era was on gut-derived putrefactive chemicals and their burden on the brain and elsewhere. As autointoxication faded away, Alvarez and others dismissed the notion that, except under rare circumstances, these chemicals could be absorbed and/or retained at any appreciable levels such that they could influence health. Although the modern gut-to-brain scientific focus has shifted to LPS and inflammatory cytokines, even the classic putrefactive chemicals (e.g. putrescine, spermidine) and some of the gut-derived and liver metabolized/conjugated structures (e.g. indoxyl sulfate, p-cresylsulfate, known classically as “ethereal sulphates”) continue to be linked to systemic cellular damage and neuropsychiatric disorders [48-52]. In particular, indoxyl sulfate, p-cresylsulfate and other uremic toxins are the subject of renewed interest as being a potential link between kidney disease and the severity of depressive symptoms. Decline in renal functioning has been consistently linked to cognitive decline and depressive symptoms. Since the kidneys are tasked with the removal of these toxins generated by colonic microbes, eliminating them via urine, it was long held that any major toxic concerns would be restricted to the later stages of chronic kidney disease.

Although the association between cardiovascular disease and kidney disease is clearly significant, uremic toxins are presenting themselves as independent predictors of CVD risk, even at surprisingly mild levels of kidney disease. Recent studies show that even mild increases in serum p-cresylsulfate (0.7mg/L) are associated with coronary artery disease [53], and experimental studies show that p-cresylsulfate causes inflammation and damage to blood vessels [54]. Moreover, experimental studies using a model of depression shows cellular damage in the renal system subsequent to psychological stress [55], meanwhile, depressive symptoms and stress have been linked to a compromise in human kidney filtration [56]. Since uremic toxins themselves can compromise kidney function, any increased burden in the load of such chemicals over a prolonged period may have profound implications in setting up a viscous cycle [57].

In animals, uremic chemicals can influence neurotransmitter levels in the brain, and emerging studies suggest they may have divergent effects on behavior depending on the level of production – in other words, too little or too much of these gut-derived chemicals may influence behavior [58]. Isatin, a chemical with similar structure to indoxyl sulfate provides an example. Compared to germ-free animals, urinary output of isatin is 50-fold higher in animals with conventional microbiota [59]. Studies show that isatin can, depending on the level, have a divergent influence on appetite, anxiety, sedation and serotonin levels in the animal brain [60]. Administration of large doses of isatin (200 mg/kg) cause a delayed increase in intestinal alkaline phosphatase (IAP) [61], suggesting it may have an influence on systemic LPS burden. Consider that a recent study indicates that there may be dozens of these biologically active colon-microbe-derived uremic solutes that remain unidentified [62]. New technologies are not only providing for proper identification, they are also allowing scientists to make previously overlooked connections between such chemicals and systemic health [63]. How these and other gut-derived chemicals might influence mental health in the context of intestinal permeability, a more porous blood–brain barrier, and low-normal kidney filtration, remains, incredibly, after the passage of a century, an open question. The antiquated urinary indican test has been replaced by profiles of urinary proteomes and metabolomic databases [64]. The extent to which probiotics and a well-diversified diet (rich in plant foods and fiber) can influence intestinal microbial ecology, the burden of putrefactive chemicals and uremic toxins, is an ongoing area of research [65,66]. The role of the liver in the metabolism of circulating putrefactive chemicals is yet another related issue.

### Additional experimental avenues

Meanwhile, other lines of experimental research continue to highlight the utility of probiotics in the prevention of intestinal permeability [67], reduction in systemic oxidative stress (inflammation and LPS load) [68,69], and in the support of nutritional pathways for optimal neurotransmitter functioning. For example, the oral administration of probiotics via laboratory chow has been shown to increase peripheral tryptophan levels [70], plasma and brain docosahexaenoic acid (DHA) levels [71,72], as well as alter serotonin and dopamine turnover in the frontal cortex and limbic system [70]. Oral probiotics appear to increase resiliency of nerve cells and reduce apoptosis during conditions of experimental physiological stress [73]. Additional research has emerged indicating that *Lactobacillus plantarum* C29, isolated from the traditional Asian food kimchi, can increase hippocampal brain derived neurotrophic factor (BDNF) [74]. Numerous reports indicate low BDNF levels in depression, and its administration in experimental models improves depressive-like behavior [75]. In experimental models of psychological stress, oral bifidobacteria reduces systemic inflammatory cytokines and normalizes brain levels of stress hormones in rats, while intentionally manipulating the diet of animals such as to double the fecal lactobacillus counts, results in decreased anxiety-like behavior [18,76,77]. In an animal model of chronic fatigue (28 day exposure to forced swim test), the administration of *L. acidophilus* (vs. placebo) reduced immobility and observations of post-swim fatigue – these effects of *L. acidophilus* were in tandem with reductions in brain measurements of oxidative stress and serum measurements of the inflammatory cytokine TNFα [78]. Recently it was reported that in addition to systemic protection against lipid peroxidation, oral *Bifidobacterium animalis* 01, isolated from centarians, decreased oxidative stress burden and brain monoamine oxidase activity, thereby potentially increasing neurotransmitter levels between synapses [79].

The influence of probiotics as a means to attenuate substance P release may also play a relevant role within the gut-brain connection. Experimental alterations to the normal gut microbiota can increase substance P release in the nervous system and promote behaviors reflective of anxiety [80]. Indeed, even very slight elevations in circulating substance P can lead to anxiety, depression and aggression [81]. Conversely, those who respond to antidepressant pharmacotherapy are known to have declines in serum substance P in conjunction with improved mood states [82]. An additional area of relevance between emerging probiotic research and depression/anxiety involves the cannabinoid receptors. Recent studies suggest that cannabinoid receptor-2 (CB-2) agonists have anxiolytic and anti-depressant properties [83]; these CB-2 agonists also limit substance P production [84] and prevent LPS-induced blood–brain barrier permeability [85]. These findings take on greater meaning given the preliminary findings regarding the ability of probiotic strains to enhance CB-2 expression [86].

Researchers have begun to explore the crosstalk biomarkers through which microbiota and host communicate. For example, *Lactobacillus plantarum* has been shown to secrete bioactive extracellular proteins capable of interacting with and modulating dendritic cells [87]. The secretion of peptides by lactobacilli, their displacement abilities relative to even minute levels of anxiety-provoking gut pathogens (e.g. *C. jejuni* as previously described) continue to be demoinstrated [88]. As researchers grapple with developmental origins of health and disease (DOHaD) [89], it is becoming clear that one important consideration may be the interaction between maternal/early life stress and the intestinal microbiota. Early life stress in animals appears to produce a more dramatic alteration to gut microbiota when acute stress is subsequently experienced in adulthood [90]. The role of neonatal exposure to probiotics in reducing the impact of subsequent adult stress on gut microbiota has recently been reported, however the extent to which this may influence behavior remains an open question [90].

In a study which takes us back to the previously described work of Fenton Turck in 1916 [91,92], a French group have recently reported that after one week of a high-fat diet, prior to full onset type 2 diabetes (or the animal model thereof), live commensal bacteria are found in the blood and adipose tissue where they are associated with low-grade inflammation [93]. The 6-week administration of *Bifidobacterium animalis* subsp. lactis 420 to these animals reversed the bacterial translocation, its associated adipose tissue inflammation and metabolic disturbances indicative of diabetes [93]. Of course, as described in Part I, these are the types of findings that were discussed, and subsequently scoffed at, during and after the halcyon days of intestinal toxemia/autointoxication. Finally, with regard to the prevention of psychological stress-induced intestinal permeability and endotoxemia, a recent study has shown that oral *L. farciminis* can reduce the effects of this vicious cycle in an animal model of acute stress [67]. Specifically, probiotic administration for 2 weeks suppressed the intestinal permeability and systemic LPS burden otherwise produced by restraint stress. Importantly, this protective quality was, in turn, associated with an attenuated hypothalamic-pituitary-adrenal (HPA) axis response and a reduction in central neuroinflammation [67]. The results suggest that, beyond direct vagal communications, the potential benefits of probiotics in mental and cognitive health, at least among some strains, may be a by-product of their ability to protect the gut lining during stress.

### Relevant clinical investigations

Recently there has been, once again, a suggestion that it might be possible to vaccinate against depression [94]. Specifically, researchers involved in a lung cancer trial using intradermally-injected heat-killed *Mycobacterium vaccae* took note of its unexpected improvement in quality of life scores. Although it did not alter cancer survival times, benefits in favor of *M. vaccae* injections included aspects of cognition, vitality, and emotional health. [95] Of course, this is reminiscent of Satterlee’s extensive writings on the use of gut microbe-derived, intradermally-injected vaccines for mental health, including his previously mentioned techniques published in JAMA [96]. It is noteworthy that *M. vaccae* is widely distributed in nature, including soil and water, and it begs the question to what extent is the withdrawal from contact with nature and its non-pathogenic microbes a factor in mental health? As mentioned, it has been recognized for over a century that lactobacilli can be found in garden soil and on plant foods [97]. Recent investigations have shown that bifidobacterium spp are also found in the soil, indeed this genera can survive in soil sampled from some of the harshest conditions on earth [98]. Very preliminary findings in animal studies show that heat-killed *M. vaccae* can beneficially influence brain serotonergic activity under stress, and live *M. vaccae* added to the mice diet improves behavioral displays of anxiety and cognitive functioning [99]. These studies indicate that a closer examination of injectable immune-modulating microbes for mental health seems warranted; indeed, as discussed later we may be forced to redefine the very term probiotic.

Human research continues to accumulate in the area of probiotics and oxidative stress. A 6-week study involving patients with type 2 diabetes showed that probiotic yogurt administration improved fasting blood glucose as well as systemic antioxidant enzyme activity and total antioxidant status [100]. In a one-month study, the administration of *L. rhamnosus* IMC 501 and *L. paracasei* IMC 502 minimized the systemic oxidative stress, lipid peroxidation in particular, associated with intense physical activity [101]. Given the documented associations between lipid peroxidation, glycemic variability and mood [102], the results are of relevance to mental health research. Moreover, a clinical study has also demonstrated the value of probiotics (*L. casei*, *L. plantarum*, *B. brevis*) in the treatment of SIBO, indeed the pilot study (n = 50) showed a more favorable response to probiotic treatment vs. the metronidazol control [103].

There has also been intriguing preliminary research involving probiotic administration and changes in the brain activity as measured via functional magnetic resonance imaging (fMRI). In a double-blind, controlled study, 45 healthy women (age 18–50) consumed either a probiotic yogurt (*Bifidobacterium lactis* CNCM I-2494, *L. bulgaricus* and *L. lactis*) vs. non-fermented dairy vs. no beverage for one month. The fMRI was conducted during an emotional reactivity task (viewing faces displaying a negative emotional reaction vs. viewing shapes). Based on blood flow assessments during the task, the probiotic group showed diminished activity in the mid/posterior insula vs. the control groups [104]. While preliminary, the results suggest that orally administered probiotics can alter brain activity in regions involved in receiving signals from the gut and the ultimate level of subsequent emotional arousal.

### Fecal microbiota transplantation

Finally, although not probiotics *per se*, a consideration of fecal transplant materials is worth mention. By the current definition, a range of live commensal bacteria from a healthy adult as used in fecal microbiota transplants (infusions), should they confer benefit, could be considered a probiotic blend of sorts. Infusion of fecal microbiota from healthy donors has recently been reported to improve insulin sensitivity in individuals with metabolic syndrome, [105] and neurological symptoms in cases of multiple sclerosis [106]. Working in reverse order, a recent case report shows major weight reduction when the opportunistic pathogen *Enterobacter cloacae* B29 was eradicated from an obese adult, and remarkably, the induction of obesity, inflammation and serum endotoxemia when that same strain was introduced to gnotobiotic mice [107]. The encouraging but very preliminary findings using fecal microbiota transplantation (FMT) will likely change the investigative path in the search for the Rosetta Stone – i.e. that which specifically defines the ideal fecal microbial (healthy donor) profile, including specific genera/species/strains, that could ultimately confer long-lasting benefit to its new host. In the meantime, a recent randomized clinical trial of *L. reuteri* ATCC55730 showed that the strain reduced mucosal inflammatory cytokine production after it was administered as a rectal enema for 8 weeks [108].

While some researchers continue to search for specific probiotic strains as transfer candidates, others are examining the use of cocktails of pathogen-free microbes derived from the entire commensal range [109]. Experimentally, in highly sensitive/anxious mice (BALB/c), the colonization of microbiota derived from more explorative NIH Swiss mice changed the BALB/c mice behavior – the typically anxious BALB/c mice became more explorative. The opposite occurred when NIH Swiss mice were colonized with the BALB/c micro biota [110]. It seems only a matter of time before FMT will be examined in those with mood and behavioral disorders where GI symptoms are present – we need no fresh hypotheses or theories to justify an attempt.

### Direct clinical investigations

The first formal investigation of the potential psychological benefits of strictly defined probiotic supplementation in humans involved 132 otherwise healthy adults; those who had more depressive symptoms at baseline had significant improvement in mood scores after taking a probiotic *Lactobacillus casei* fermented beverage compared to the placebo group [111]. A separate placebo-controlled pilot study involved 39 chronic fatigue syndrome patients who were administered the same oral *Lactobacillus casei* probiotic vs. placebo. At the conclusion of the 8-week study, depression scores remained unchanged between the groups, however there were significant improvements in anxiety as measured via the Beck Anxiety Inventory vs. placebo [112].

Even more recently, French researchers evaluated a *Lactobacillus helveticus* and *Bifidobacterium longum* combination probiotic which was orally administered for one month in a placebo-controlled study. Using a variety of validated anxiety, stress, and depression scales, researchers reported significant improvements in day-to-day depression, anger, anxiety, as well as lower levels of the stress hormone cortisol among otherwise healthy adults taking a daily probiotic supplement vs. placebo. In addition, an experimental arm of this study also confirmed that the probiotic added to the chow of rats did indeed decrease behaviors indicative of anxiety [113]. Moreover, given the above-mentioned study linking baseline depressive symptoms and improved mental outlook among healthy adults after probiotic administration, the French group performed a secondary analysis looking specifically at those with the lowest baseline urinary free cortisol (n = 25). Indeed, the results once again showed improvement with *Lactobacillus helveticus* and *Bifidobacterium longum* vs. controls (particularly in somatization, depression and anger-hostility), and among this low cortisol sub-group the overall benefits in anxiety and depression were pronounced over time [114]. Finally, in a study involving 44 patients with irritable bowel syndrome, the oral consumption of a prebiotic fiber (trans-galactooligosaccharide) significantly reduced anxiety in conjunction with marked elevations in fecal *bifidobacteria* levels [115].

## Conclusions and future directions

Looking back at the first three decades of the 20th century is an experience, for the modern scientist interested in the gut-microbiome-brain connection, akin to an anthropological archeologist discovering an ancient civilization. Superficially, the historical markings are dominated, as they often are in medicine, by the myopic and biased view toward the so-called ‘great/infamous men, great/infamous discoveries and claims’. Medical historians focus on heroes and anti-heroes in the fights against ignorance and disease. Yet the complete chronicles are never as simple as Lane vs. Alvarez. Psychiatrist and medical historian Iago Galdston warned against losing context and understanding with such chronicles - ‘*The essential deficiencies in academic medical history derive from its commitment to the “great man, great discoveries” view of medical history and of medical progress*…*those who labour long at such gathering are prone to mistake their miscellany of accumulations for real knowledge and deep understanding*’ [116]. Sifting through the archives, moving past the outer walls of intestinal autointoxication dominated by Metchnikoff, Lane, Cotton and non-physician opportunists, one finds rational works that were in line with modern-day ‘discoveries’ - antecedents to what we are now, in many ways, relearning and constructing related to the importance of the gut integrity and its microbial residents. An examination of the early history of oral bacteriotherapy reveals hundreds of studies dedicated to the intestinal flora and its transformation. Although many of these studies reported conflicting findings, and the methodologies were most certainly based on rudimentary technique by the standards of today, there were more than enough clues to suggest that the primary premise – i.e. gut-derived microbes and/or microbial breakdown products *may play a role* in mental health – was correct.

Related concerns of intestinal permeability, SIBO, hypochlorhydria, carbohydrate intolerance, endotoxins, modernity and dietary matters, gut microbe vaccines, the oral administration of lactic acid bacteria, colonic microbiota transfer, and qualitative/quantitative changes to the intestinal microbiota were all discussed as being relevant to mental health and cognition. For a variety of reasons, not the least of which included lack of human evidence and broad claims that autointoxication was the exclusive root of all neuropsychiatric disorders, these discussions disappeared from mental health publications. One cannot say that the disappearance of autointoxication was driven exclusively by the emergence of evidence-based medicine because in many cases it would be supplanted by unverifiable Freudian psychoanalytic viewpoints. Still, clinicians pleaded for more than studies in rodents, rabbits and monkeys – they needed convincing human research so that medicine could be practiced not by hypothesis - and yet none would be forthcoming. Unwarranted colectomies and unfounded marketing promissory notes for good health via friendly microbes would obscure legitimate research pathways that might have otherwise been followed with vigor. They also created a superficial and simplified medical history based on great or infamous men. Over time, autointoxication would become known only for these surgical and marketing extremes, ultimately becoming a medical outcast – or, as it was written just prior to our hypotheses papers, ‘*a triumph of ignorance over science*’. Yet, as the French physiologist Claude Bernard wrote, “*That which we know is a great hindrance to our learning that which is yet unknown to us*”. By the year 2000 we “knew” intestinal toxemia and friendly microbes for mental health to be exclusively a medical folly, one associated with charlatans and so-called colon cleansers.

All of this contemporary work, as described above, forces us to have a fresh look at the inner dialogue within the historical intestinal toxemia publications. Obviously the modern advances are not a validation of colectomy, *L. bulgaricus* as a fountain of youth, or colon cleansers as a solution to all the potential ills of what may indeed be a type of intestinal ‘toxemia’. However, modern historical reviews that stick exclusively to the redundant and superficial narrative of “autointoxication was the arena of charlatans, it was wrong and disproven by Alvarez” contribute little, and indeed provide a disservice to the medical minds that were on a rational path. Of course, reminders of the dangers of practicing medicine by hypothesis alone have their place. However, it may be time to re-write some of the history books or at least qualify them; the legacy of Lane and Cotton should not negate that of those who found that a high fat diet and stress increased intestinal permeability and bacterial translocation, or those who reported successful outcomes with simple fecal transplantation. Why should the legacy of those who instilled fears into *healthy* adults regarding autointoxication, as a means to sell pseudoscientific contraptions, obscure that of rational physicians who saw some legitimacy to intestinal toxemia as relevant to *unhealthy* populations? It is fairly obvious many of those physicians rationally discussing intestinal toxemia a century ago were confronted with unhealthy patients (IBS, ME, FM, migraine, mood and anxiety disorders) that would today be classified with elaborate diagnostic criteria and codes.

Top-down, psychosomatic-oriented theorists [117] continue to haul out Lane and Cotton as exhibits A and B to exclaim that autointoxication as it relates to mental health was nonsense [118] - with nary a mention of the history of the other not-so-Great Men, and the emerging gut-brain-microbiome research. Psychosomatic researchers continue in their quest to show that IBS is provoked by neuroticism – non-prospective, x-sectional population studies show high degrees of neuroticism correlate with IBS severity, and the Pubmed abstracts boldly conclude that ‘*These results suggest that neuroticism is involved in the pathophysiology of IBS*’ [119]. These strong assertions are accompanied by nary a mention of gut microbiota. What if it were the opposite? What if the bottom-up microbiota is involved in the provocation of neuroticism among IBS patients? Or what if, as is more likely the case, it lays somewhere in the middle ground? Once again, psychiatrist and historian Iago Galdston, provided advice for research and direction (in 1954) of what he hoped would be a new era in psychosomatic medicine, one without labels of “type” (e.g. Alvarez’s go-getter “ulcer” type etc.) as they pertain to disease. He advised a shift to the middle ground – ‘*we will learn to understand the experiences of man in terms of multidirectional relations, and with a simultaneity that is free of the naiveté and artificiality of straight line sequential causality…when we have come to such an understanding, psychosomatic medicine will be truly holistic. But then, may I whisper it softly, it will no longer be psychosomatic medicine. It will be medicine such as Hippocrates could comprehend, and Paracelsus might celebrate – a keen reflection on the interrelations of Microcosm and Macrocosm*’ [120].

With all our modern advances, we once again arrive at a place of theory, albeit slightly more sound in its support. In order to truly advance from the days of Metchnikoff we must expand the bench and rodent work and bring it into the clinical investigative setting, and not to do so, of course, for the purpose of yet more anecdote. The undoing of intestinal toxemia and the idea of probiotics for brain health was not Alvarez, it wasn’t the charlatans and the high colonics, nor was it for the want of more rodent and laboratory studies - its undoing was the lack of convincing controlled clinical work. It seems remarkable, given the supporting scientific rationale, that there has been so little in the way of clinical research. At this point, as highlighted throughout this series, we already have extensive experimental research from which to guide probiotic strain selection for clinical experimentation – e.g. strains that can lower LPS burden; strains that lower oxidative stress, and systemic inflammatory cytokines; strains that have a beneficial influence on stress resiliency; strains that can influence neurotransmitter precursor levels via amino acids and neuronal membrane structure via fatty acids; strains that can attenuate intestinal permeability; strains that can lower uremic toxin burden. It may take decades to further elucidate the divergent ways in which these and other specific probiotic strains may interact, alone and in combination with each other, to influence markers relevant to animal models of depression and/or anxiety. This critical work should, of course, continue. However, the mouse models will always be lacking the clinically-relevant context of mood-related diet, physical activity, environmental toxin exposure and other variables within lifestyle medicine. Probiotics, even if they do influence the *human* GABA system, are not synthetic benzodiazepines; as discussed throughout this paper, orally administered microbes are much more likely to converge with lifestyle variables within the gut lumen.

This presents a quandary for experimental researchers examining the use of probiotics in the behavior of animals as a means to inform clinical utility. Since environmental enrichment factors are known to interact with psychological stress and diet, both of which interact with gut microbiota, a near-endless combination of animal housing and dietary variables related to microbes must be investigated - from running wheels, tunnel positioning, wood, plastic and soil materials [121-123]. To put it more clearly, a high-fat diet can compromise brain function as previously described, however, the detrimental cognitive effects of too much fat can be significantly offset by the degree of naturalistic environment [124] in which the animals reside! Could there be probiotic strains that might appear to be without value in one model, yet provide value when interacting with environmental variables such as physical activity? As for diet itself, a multitude of dietary variables could potentially interact with probiotic administration in animal behavioral studies. The potential value of a specific strain or groups of strains of probiotics may be dependent, or put another way, may be obscured, by a host of dietary variables. Despite more than ample scientific justification, only our own group [112] and a few others have begun to explore the potential of probiotics to influence *human* mood, cognition and fatigue. The results from these early studies, in concert with what is now a fairly robust body of experimental research, would suggest that we have now passed the time in which clinical investigations should be approached with vigor. Moreover, the work of Michaël Messaoudi [113,114] and colleagues from France deserve special accolades, for this group has been simultaneously sampling the effects of probiotcs on behavior in animal models and mental health in clinical settings. Human intervention studies with probiotics related to allergy risk, including prenatal/early life administration, have been ongoing for more than a decade – perhaps it is time for neuropsychiatric researchers to catch up.

The strain specific focus is clearly justifiable in the scientific examination of a particular probiotic preparation or commercial ‘functional food’ for mental health. Research not only shows that heat-killed bacteria may have strain-specific benefit, the research also indicates that there are broad and divergent effects of lactic acid bacteria in their interaction with the immune system [125]. If we isolate single strains, we should indeed have a good degree of certainty that its potential influence is that which is desired in immune function over the long term. Less known is the extent to which an individual strain might influence the overall microbial ecology of the gut when consumed for extended periods of time. Could a single strain diminish diversity? The isolated strain approach also carries a less speculative caveat; it tells us little about the potential synergistic benefits of multi-species lactic acid and other bacterium found in traditional diets and fermented foods. Are we obscuring microbial benefits with a myopic view to single patented and/or commercial strain? Beneficial microbes are abundant in indigenous diets, and an estimated 35% of all lactic acid bacteria isolated from raw fruits and vegetables can survive gastric conditions [126]. Beyond lactic acid bacteria we can consider the previously mentioned studies on live *M. vaccae* added to the dietary of animals – as a soil-derived organism, *M. vaccae* can easily find its way onto plant foods. It seems the links between traditional dietary patterns, mental health and microbiota, are far more complex than generally appreciated. We simply cannot view the gut-brain-microbiota axis as isolated from diet, the context of its macro and micronutrient as well as phytochemical composition. This fact underscores the potential futility of probiotic administration to a sedentary individual with depression that may be co-consuming a fast-food style diet with the addition of pharmaceuticals and/or dietary chemicals (e.g. sucralose) that are otherwise capable of altering the intestinal microbiota [127]. These are just some of the clinical realities not addressed in simple experiments with rodents in an elevated plus maze.

### Have we advanced, will we advance?

In the real-world of mental healthcare provider and patient, one that occurs in a holistic setting involving lifestyle factors, the odds of a single strain of probiotic bacterium providing clinically meaningful and long-lasting benefit, not simply statistically significant differences vs. placebo, could not be estimated to be high. Yet, probiotic experiments with rodents, just as they did in the day of Metchnikoff (“Yoghurt – the anti-toxin of old age” May, 1913) [128], generate headlines such as “Forget Prozac – Try Probiotics” (Sept 10, 2012) [129]. Widely disseminated inferences that, based on current rodent research, probiotics are a substitute for fluoxetine are not only alarming, such headlines should ultimately force us to ponder to what extent we have truly advanced from Metchnikoff.

Today, in the new era of Autointoxication II, what is old is new again – preliminary non-clinical research efforts, based on metagenomics and microbiome projects, are commercially co-opted to support a financially lucrative probiotic business wherein unsubstantiated claims abound [130]. Metchnikoff’s history is repeating itself - in a 2011 study published in *PLoS One*, Japanese researchers showed that a strain of *Bifidobacterium animalis* can increase longevity in mice via its influence on gut polyamine production [131]. In 2012 researchers linked negative mood, social anxiety and distressed personality type, if you can imagine, to indoxyl sulfate levels in a healthy (n = 1502) population [132]; meanwhile, in other news, there are now commercially available probiotic formulas in North America that are clearly positioned, in their direct product names and associated claims, as anti-aging and anti-stress formulas [130]. It seems fair to ask, at this juncture, when will this research pathway truly enter and *emerge* from phase I of translational medicine? Will all of this work become another clinically meaningless forgotten city in the future, one destined to once again be explained away by conflicts over toilet training? If there is a future role of probiotics in cognitive and mental health, one relevant for clinicians, and one that can only be proven or disproven by human intervention studies, it is almost certainly as our group hypothesized it to be – an adjuvant to well-established first-line care.

## Competing interests

ACB and EMS have no competing interests. ACL has received consulting fees from Genuine Health, Toronto, Canada.

## Authors’ contributions

ACB, ACL and EMS contributed equal time and effort in the investigation, research and drafting of this manuscript. All authors read and approved the final manuscript.
